# Current practice in prescribing footwear and insoles to reduce the risk of neuropathic plantar forefoot ulceration and re-ulceration in people with diabetes

**DOI:** 10.1371/journal.pone.0341594

**Published:** 2026-02-10

**Authors:** Sayed Ahmed, Alex Barwick, Anita Sharma, Md Zobaer Hasan, Muhammad Ashad Kabir, Kunal Kumar, Susan Nancarrow

**Affiliations:** 1 Faculty of Health, Southern Cross University, Lismore, Queensland, Australia; 2 Foot Balance Technology Pty Ltd, Sydney, New South Wales, Australia; 3 Department of Geriatric Medicine, Nepean Hospital, Penrith, New South Wales, Australia; 4 Faculty of Medicine and Health, Nepean Clinical School, University of Sydney, Camperdown, New South Wales, Australia; 5 School of Science, Monash University Malaysia, Jalan Lagoon Selatan, Bandar Sunway, Selangor Darul Ehsan, Malaysia; 6 School of Computing, Mathematics and Engineering, Charles Sturt University, Bathurst, New South Wales, Australia; Polytechnic University of Marche: Universita Politecnica delle Marche, ITALY

## Abstract

**Background:**

Footwear and insoles are frequently used to offload the neuropathic diabetic foot at risk of ulceration and re-ulceration. This study aims to explore the prescription habits of Australian pedorthists and the variations in footwear and insole design due to variations in foot pathology, comorbidities and patient-specific factors consideration.

**Methods:**

An online survey link was sent to Australian pedorthists (n = 42), who were asked to answer questions relating to four hypothetical cases exploring current footwear and insole prescribing habits. Qualtrics research and data analysis software was used for the online survey and data analysis tool.

**Results:**

Custom-made footwear was commonly prescribed in more complex cases, with recommendations ranging from 47% to 80% across scenarios (e.g., 80% in Case-4). In simpler cases, prefabricated medical-grade footwear with modifications was more frequently recommended (e.g., 53% in Case-1). Custom-made insoles were recommended by 84% to 100% of participants across all cases. Variations in prescriptions were largely influenced by differences in pathology, comorbidities, and individual patient preferences. Common barriers to patient adherence included concerns about appearance, bulkiness, and difficulty putting on or removing the footwear. To address these, pedorthists employed strategies such as involving family members in design choices, offering dual-purpose footwear (e.g., for indoor and outdoor use), incorporating culturally or gender-sensitive designs, and using in-shoe pressure mapping to demonstrate clinical benefits.

**Discussion and conclusion:**

Australian pedorthists follow national and international guidelines for footwear and insole prescriptions with some variations when they encounter patient-specific factors such as preferences and lifestyle. The majority of them follow the best practices when evaluating the efficacy of the recommended devices, such as in-shoe pressure mapping systems. They also employ various practical techniques to increase adherence towards improved patient outcomes by engaging family or partners and through motivational interviews and interdisciplinary team approaches.

## Introduction

Foot ulcers are a common consequence of diabetes due to the development of peripheral neuropathy, peripheral vascular disease, limited joint mobility and foot deformity [1–6]. Up to 34% of people with diabetes will develop a foot ulcer in their lifetime [7]. These foot ulcers can lead to infection and amputation, with diabetes being the main reason for non-traumatic amputation [8]. Furthermore, those with a previous foot ulcer or amputation are at high risk of future amputation [1,3,5,9].

Neuropathy is a crucial factor in the development of ulceration [10]. The most common neuropathic ulcers in diabetic feet are in the plantar forefoot area [11,12]. Over 90% of foot ulcers are located at the forefoot plantar region [13], corresponding to sites of peak plantar pressure [14]. This is, therefore, a key site for preventative interventions, including custom accommodative orthoses and therapeutic footwear.

There is evidence that wearing certain footwear can reduce and prevent foot ulceration by redistributing areas of peak plantar pressure [15,16]. Footwear is a complex biomechanical intervention due to the large variance in design, materials, manufacturing methods, and individual preferences and rates of adherence. It is widely considered that the attributes of shoes that need to be considered for people with diabetes are shoe length, depth, width, height, insole, outsole, rocker profile, heel enclosure, heel lift or pitch, closure, uppers and toe box [17,18]. However, the footwear parameters most important for the prevention of ulceration at these sites require further research.

The complexity of footwear design is compounded when it is considered alongside the range of foot pathologies that co-exist with diabetes. Severe structural deformities, including hammer and clawed toes, hallux valgus, and crossover toes, are prevalent in this patient group [12,19]. This study aims to explore the current practice in footwear and insole prescription, design and manufacturing for people with complex foot complications commonly seen in pedorthic practice.

Pedorthists commonly prescribe and fabricate custom-made diabetic footwear or modify stock diabetic shoes and accommodative insoles for this population. There are three key stakeholders related to diabetic footwear practice. These include referrers, prescribers and consumers. Patients are commonly sent for pedorthics management with a formal referral letter and federal or state-supported funding, such as the funding available through Enable NSW, [20] NDIS, DVA. Importantly, the high-risk foot service standards recommended by NADC [21] support these referral pathways.

Cavanagh and colleagues [22] demonstrated a gap between evidence-based guidelines and current clinical practice in diabetic foot offloading. Hence, this study aimed to explore the current practices by pedorthists to improve offloading at plantar forefoot regions in patients with diabetes and neuropathy.

## Methods

### Sample

The target population for the survey study were all Australian pedorthists registered with the Australian Pedorthists Registration Board (APRB) [23]. Searches through the PAA [24] and APRB [23] websites were performed, identifying all registered pedorthists. During the study period, 42 certified pedorthists practising in Australia were registered under the APRB, [23] and all pedorthists were sent an invitation to participate.

### Case Study development

A case study is typically an intensive analysis of an individual unit (as a person or community) stressing developmental factors in relation to the environment.

Clinical cases are typically used in clinical education.

For the purpose of this study, clinical cases were developed to represent ‘typical’ patients that might be seen in clinical practice to provide a focus for understanding the breadth and depth of variation in the prescribing practices of Australian pedorthists. The clinical audit [25] data were used to help understand the attributes of the ‘typical’ patient. A series of case studies were developed with some variation for gender and cultural diversity (including some minor cultural group subjects to increase representability) and further refined in consultation with an expert panel (high-risk foot clinics podiatrists and senior pedorthists). The result was four cases from four unique culturally diverse backgrounds to capture the variations for socioeconomic, health fund eligibility and activity-specific factors. Table 1 presents the summary of four hypothetical cases developed as an outcome of this study and to be used as baseline information for the Australian pedorthists survey (This study). Additionally, Table 2 presents a summary of cases developed for the survey of Australian pedorthists.

**Table 1:** Summary of justifications for the case parameters included in the cases developed for the survey of Australian pedorthists.

Parameter	Attributes proposed for the cases	Justifications
**Gender**	Female X 2, Male X 2,	Gender was evenly split between men and women, although the data showed more men are at risk in this group.
**Age**	55-70 years,	The average age range in the literature is 63 ± 10 years for this population, and our study is 64.09. Our range reflects this.
**Height**	170-178 cm	
**Weight, BMI**	76-116 kg, 26.3–39.2,	In the literature, the population BMI for men and women range is 30.04 ± 6.09, and our audit and range reflect that
**Cultural diversity**	Caucasian, Australian Aboriginal, Fiji Indian, Chinese	In the literature and our audit, reflect the same as our range
**Living arrangements**	Private home, alone, with husband, with daughter,	Data obtained from our audit and our range reflect that
**Activity level**	Active, low activity and minimal activity, smoker	In the literature and our audit, reflect the same as our range
**Care Support**	None to Three days/per week	Data obtained from our audit and our range reflect that
**Duration of Diabetes**	Between Ten to 39 years	In the literature and our audit, reflect the same as our range
**Duration of Neuropathy**	Seven to ten years	In the literature and our audit, reflect the same as our range
**Comorbidities**	PVD, Oedema, Nephropathy, Hypertension, Rheumatoid arthritis, Retinopathy	In the literature and our audit, reflect the same as our range
**History of Foot Ulceration/** **amputation**	R plantar hallux ulceration Nil amputation, L plantar metatarsophalangeal joint three Hallux amputation R, R plantar metatarsophalangeal joint one L Transmetatarsal amputation, R plantar metatarsophalangeal joint L D3 amputation Achilles tendon lengthening (6/12 prior)	In the literature and our audit, reflect the same as our range
**Hyperkeratosis location**	Dorsal digits two and three, Lateral left D5, Nil pronounced, Severe plantar L D4, D5	In the literature and our audit reflect the same as our range
**Foot morphology**	Bilateral bony prominences MTH1,5 Lesser digit hammertoes HAV, B Rigid pes cavus, L Adductovarus 5 Lesser digit Hammertoes R > L, L Hallux limitus B Rigid Flatfoot, R Over-riding D2 on D3	In the literature and our audit, reflect the same as our range

**Table 2:** Summary of cases developed for the survey of Australian pedorthists.

Parameter	Case-1	Case-2	Case-3	Case-4
**Gender**	Female	Male	Male	Female
**Age**	65 years	55 years	70 years	55 years
**Height**	170 cm	178 cm	172 cm	170 cm
**Weight, BMI**	86 kg, 29.8	98 kg, 30.9	116 kg, 39.2	76 kg, 26.3
**Cultural diversity**	Caucasian	Australian Aboriginal	Fiji Indian	Chinese
**Living arrangements**	Private home, with husband	Private home, alone	Community housing, alone	Private home, with her 30 y/o daughter
**Activity level**	Active	Active, smoker	Low activity	Minimal activity
**Care Support**	None	None	Three days/week	None
**Duration of Diabetes**	Ten years	Ten years	18 years	39 years
**Duration of Neuropathy**	Seven years	Five years	12 years	Ten years
**Comorbidities**	Peripheral vascular disease Oedema	Retinopathy Hypertension	Oedema Nephropathy Hypertension	Rheumatoid arthritis Retinopathy Hypertension
**History of Foot Ulceration/** **amputation**	R plantar hallux ulceration Nil amputation	L plantar metatarsophalangeal joint three Hallux amputation R	R plantar metatarsophalangeal joint one L Transmetatarsal amputation	R plantar metatarsophalangeal joint L D3 amputation Achilles tendon lengthening (6/12 prior)
**Hyperkeratosis location**	Dorsal digits two and three.	Lateral left D5	Nil pronounced	Severe plantar L D4, D5
**Foot morphology**	Bilateral bony prominences metatarsal heads one and five Lesser digit hammertoes Hallux abductovalgus	B Rigid pes cavus L Adductovarus 5 Lesser digit Hammertoes R > L	L Hallux limitus B Rigid Flatfoot	R Over-riding D2 on D3 B hallux abductovalgus

### Survey development

A survey questionnaire was designed to evaluate the current practice of footwear and insoles prescription by certified pedorthists in Australia. Four hypothesised ‘typical’ or ‘illustrative’ cases were developed to evaluate common practice. The development of these cases is outlined in S1File (Appendices). The survey included multiple-choice questions and text input options as required.

The questionnaire had three sections:

1) Section 1 – case presentations: presentation of the hypothesised patients’ diagnosis, comorbidities, psychosocial factors and sociodemographic information. These were formed by the findings from the clinical audit [25].
2) Section 2 – footwear prescription: questions regarding the footwear the participant would prescribe for each case. This included footwear type, upper design, upper materials, heel height, toe spring, heel counter, opening, fastening, modifications, rocker parameters, and sole materials. This included multiple-answer questions and open-text comments. The questionnaire also prompted respondents to outline challenges they would face while recommending therapeutic footwear from patients’ adherence perspectives and what strategies they would consider overcoming those challenges.
3) Section 3 – insole prescription: Questions regarding the insoles the pedorthists would prescribe for each case. This included insole type, casting method and materials. This included multiple-answer questions and open-text comments. The questionnaire prompted respondents to outline challenges they would face while recommending insoles from patient adherence perspectives and what strategies they would consider overcoming those challenges.

Following this, a multiple-choice question was asked on how the pedorthists would measure the offloading success of the prescribed footwear and insoles.

The full questionnaire is available in Appendix in S1 File.

An expert panel, comprising three senior Australian pedorthists with over 20 years of clinical experience, piloted this draft survey tool. In addition, the pathology and comorbidity sections of the questions were verified by five senior podiatrists, each of whom has more than five years of experience working in high-risk foot clinics in public tertiary hospitals. They reviewed the contents of cases and questions to ensure sufficient variety in the cases to elicit the full variety of practice and for the authenticity of the cases. A summary of the cases is provided in S1 File.

The attributes included in sections 2 and 3 of the questionnaire were derived from the systematic literature review conducted by the researcher [18] and Diabetes Feet Australia (DFA) guidelines [17] on footwear for people with diabetes. These documents outline the key recommendations on footwear type, footwear upper design, rocker sole design profiles, insole characteristics such as insole type, casting methods, insole materials, various components of the insole design feature, and patient adherence-related information.

**Fig 1:**
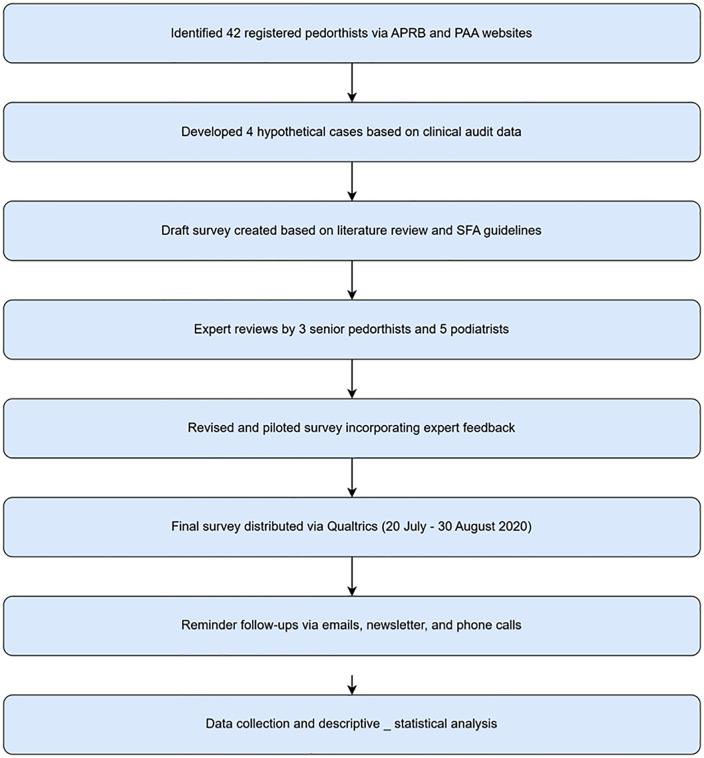
Flow diagram of survey development and distribution.

The expert panel piloted the full questionnaire, and their suggestions were incorporated into the survey tool for the final version. Based on the feedback of the expert panels, the diagnosis and comorbidity domains (section 1) were simplified, and the information on the footwear and insole prescriptions to allow participants to enter commercial names of the materials was added. The flow diagram for the survey development and distribution is shown in Fig 1.

The psychometric properties of the survey tool was used to address multiple forms of validity. The tool established content validity via expert panel review which engaged three senior pedorthists with over 20 years of experience and five podiatrists with over 5 years’ experience in high-risk foot care. The experts assessed the survey for relevance, accuracy, and completeness. Face validity was assessed by the tool via pilot testing and the same expert panel, who reviewed the results of the survey for clarity and clinical relevance. Their feedback was used to enhance the usability on the interventions. Construct validity was supported by grounding the survey content in a prior systematic literature review and in line with the DFA guidelines that informed attributes related to footwear and adherence. The study did not conduct any formal reliability testing and this is acknowledged as a limitation of the tool.

### Data collection

The survey questionnaire was distributed via email using an online survey tool, Qualtrics, [26] to the pedorthists who were registered with APRB. The online survey was open for the pedorthists to respond to from 20 July 2020–30 August 2020.

### Data analysis

A descriptive analysis of footwear practice recommended by the participants was undertaken, including footwear type, upper design, type of opening and fastening, type of insole and casting methods for each of the four cases. Furthermore, a descriptive analysis was performed on the following parameters for footwear features: heel height, toe spring, upper, lining, padding between upper and lining, reinforcement and heel counter, rocker sole design parameters, bottom construction materials (midsole, outsole, sole and wedge, heel), insole/orthoses design materials (insole base, mid-layer and top cover), other insole/orthoses design/modification features (additional arch support, metatarsal dome, metatarsal bar, metatarsal pad and local cushioning) for all four cases. Furthermore, an evaluation of how offloading success was evaluated for all four cases was undertaken. Also, a statistical test (Fisher’s Exact Test) was conducted to investigate the association between the casting method of making insoles and the sociodemographic characteristics (Case, Gender and Age Group).

The frequency of each of the above-listed manufacture characteristics for footwear and insoles was calculated. Qualitative information from open text questions related to how adherence was managed and challenges faced were integrated and categorised into common themes and presented through various graphs such as scatter plots, grouped bar charts, stacked bars and funnel charts.

### Ethics approval and participants’ consent

Southern Cross University Health and Human Research Ethics Committee gave the Ethics approval for this study, and the Approval number is 2020/028. Written informed consent was obtained from all participants prior to their participation through the online questionnaire. An information sheet outlining the study’s purpose, procedures, and confidentiality assurances was provided, and consent was electronically recorded when participants agreed before commencing the questionnaire. The study did not include any minors.

## Results

### Participants

At the time of the survey, 42 pedorthists in Australia were eligible to participate in this study. Overall, 19 pedorthists out of 42 responded to the online survey (45% response rate). Two follow-up reminders were sent from the survey database to those who were sent the initial survey participation invitations and had filled out questionnaires partially. Reminders were also sent to the pedorthists through the monthly newswire of PAA and some personal phone calls and emails by the PAA executive officer to the members to increase the survey participants of the pedorthists.

Participating pedorthists were eligible to receive continuing professional development points as a result of their participation in this study.

Of 19 participating pedorthists, at least ten pedorthists answered all four case questionnaires. In total, 19 participants responded to Case-1. Fewer pedorthists responded to Cases 2, 3 and 4 (n = 11 (26%), 10 (24%) and 10 (24%)). The ratio of the responses to each case was expected considering the complexity level of the cases that relate to the skills level and service offering capacity.

### Footwear characteristics

The footwear characteristics of interest were footwear type, upper design, fastening system, heel height and toe spring, heel counter, materials for upper and lining, and footwear modifications to improve functions and usability. There were high levels of agreement in terms of upper-type, fastening systems and footwear modification recommendations for individual cases, but a great deal of variation around heel height and toe spring recommendations for individual cases. More consistency was seen for cases 3 and 2 and less for cases 1 and 4 in terms of the footwear characteristics recommendations.

**Fig 2:**
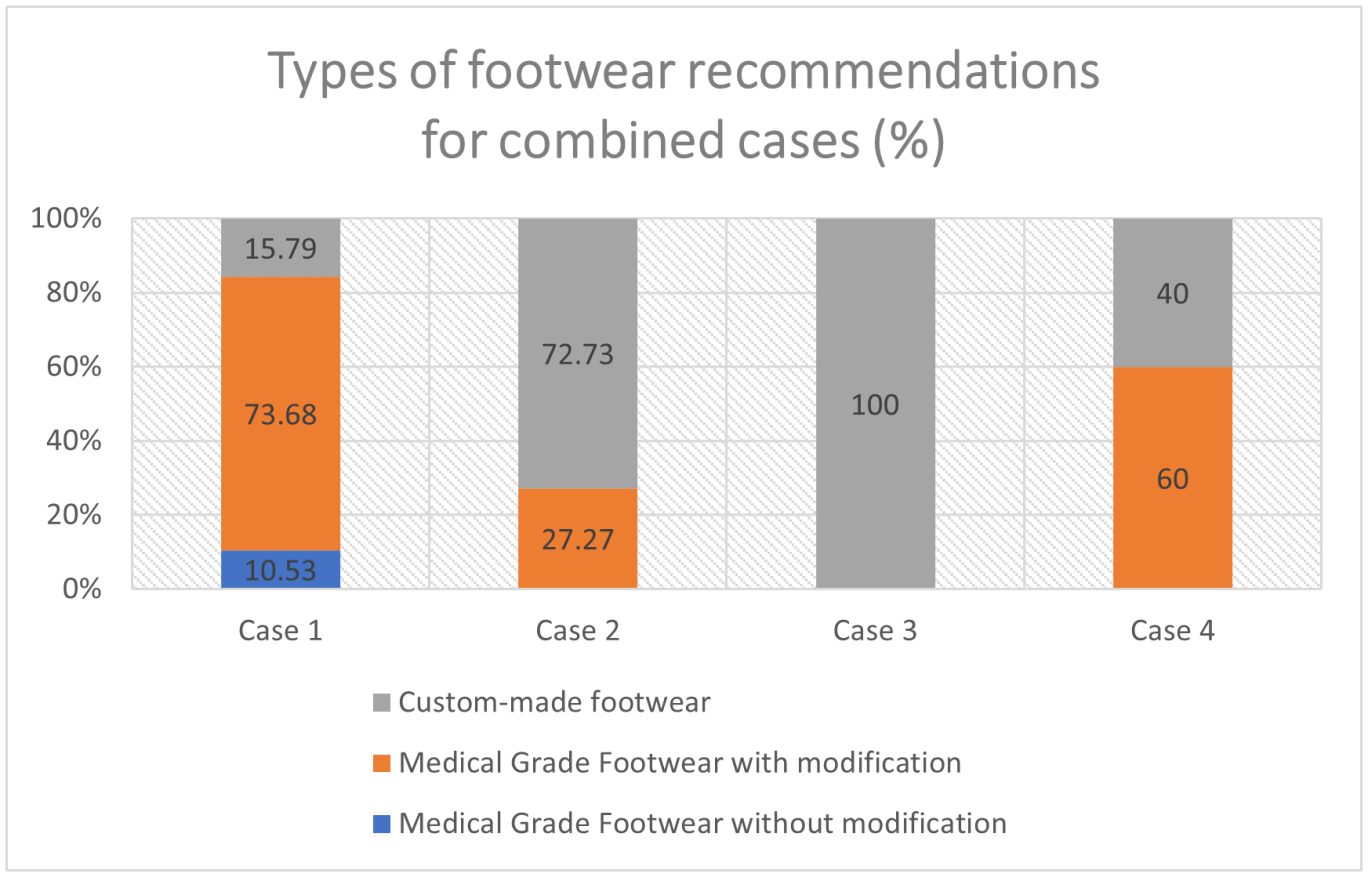
Types of footwear recommendations for combined cases.

These are outlined in more detail below, with Fig 2 displaying the proportion of respondents recommending each overall footwear type (custom-made footwear or medical-grade footwear with or without further modification) for each case.

### Upper design

Across all four cases, clinicians most frequently recommended the “Bottine (mid-height)” footwear, with approximately 20–25 respondents selecting it for each scenario. The “Lowcut” and “Above ankle boot” options were consistently less favored across all cases, generally attracting fewer than 10 respondents each. This pattern suggests a clear preference among clinicians for mid-height footwear regardless of the specific case presented. Furthermore, Fig 1 shows the variation in recommendations from respondents regarding upper design, including overall design (low, bottine and above the ankle upper) and the degree (cm) of upper heights specified, respectively.

#### Heel height.

The recommendation by the pedorthists for heel height varies between 1 cm to 3.5 cm. Most respondents recommended around 2 cm heel height (n = 7 for Case-1, n = 6 for Case-4) for women’s shoes, whereas around 1 cm height was the second most recommended (n = 5 for Case-1, n = 4 for Case-4).

**Fig 3:**
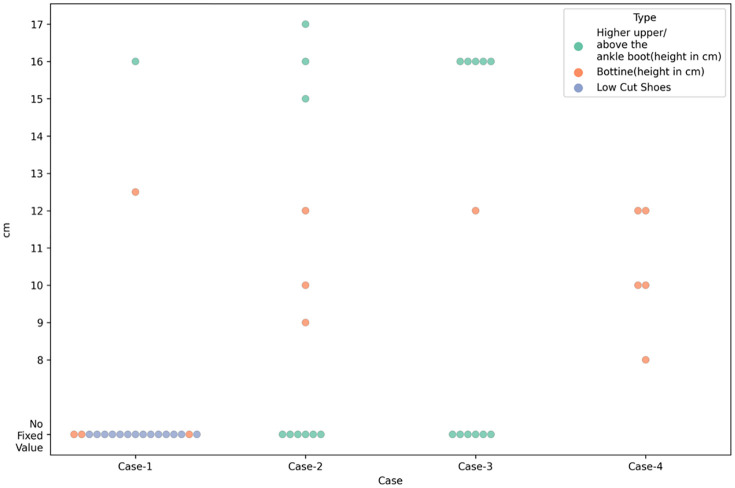
Upper heights recommendation for Case-1, Case-2, Case-3, and Case-4.

For men’s shoes (Case-2 and Case-3), the commonly recommended heel heights by the pedorthists are around 1 cm, followed by around 2 cm heel height. Toe spring recommendations vary a lot, from 0.4 cm to 3 cm. Fig 3 presents the heel height recommendations for the cases.

Some participants commented on the balance issue of the person, and some tried to combine the rocker angle and insole profile to determine the final functional toe spring. Fig 5 illustrates the apex angle recommendations while Fig 6 display the rocker angle recommendations.

**Fig 4:**
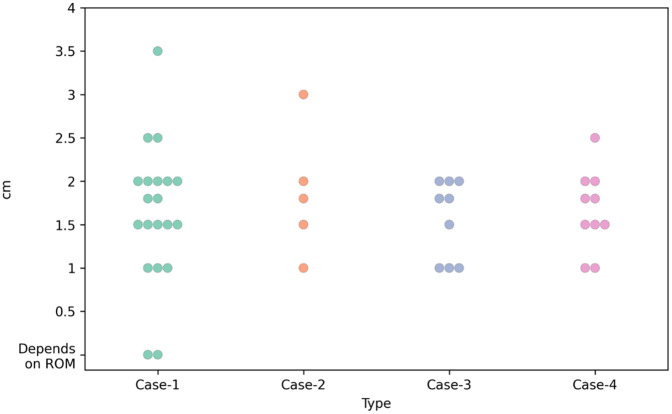
Heel height recommended for Case-1, Case-2, Case-3 and Case-4 (scatter plot).

1 cm toe spring has been mostly preferred by the pedorthists for Case-1 (n = 7) and Case-4 (n = 4). For Case-2 and Case-3, the recommended toe springs are a little higher due to the lower heel height recommendations because heel height and toe spring are recommended in reverse. The detailed recommendations on toe spring are presented in Fig 4 for all four cases. The detailed information on recommendations for heel counters by pedorthists is in Table 3.

**Table 3:** Footwear design recommended for Case-1, Case-2, Case-3 and Case-4.

Parameters for footwear features	Case-1		Case-2		Case-3		Case-4	
	Description	n	Description	n	Description	n	Description	n
**Heel counter type**	Standard	12	Standard	2	Medial extended and reinforced	2	Medial/ lateral extended	1
	Extended medial	1	M & L elongated	1	Standard	2	Standard	6
	Lateral extended	1	Reinforced		Medial extended	2		
	Medial/lateral extended, reinforced	1	Reinforced extended medial/lateral	1	Extended medial& lateral	1		
	Medial extended	1	Lateral extended +reinforced	2	Medial Reinforced	1		
	Standard medial	1			Extended	1		
**Type of opening and fastening**	Neoprene padded topline	1	As an alternative to only lace-up	1	Medial zip	1	Forward opening	1
					Forward opening to aid easy access for the rigid flat foot into the shoes	1	Velcro selected due to RA may affect the hands	1
**Upper materials**	Leather	7	Combination of leather and fabric (breathable, if possible, like Gore-Tex)	1	Leather	8	Stretchable leather, neoprene, material	1
	Soft leather	3	Soft but durable Leather	3	Leather/ neoprene	1	Lycra stretch/leather combo	1
	Neoprene/ stretchable/ Lycra	1	Leather	11	Soft leather	1	Leather	2
	Neoprene & leather plug in the vamp	1					Soft leather	4
							Leather/neoprene	3
Lining materials	Leather	7	Leather	1	Leather	4	Soft leather with no seams in the toe area	3
	Diabetic lining Airnet	1	Leather Calf medium-weight	1	Leather/ neoprene	1	Stretchable leather, neoprene, material	2
	Soft non-leather	1	Diabetic lining Airnet	1	Leather seamless	1	Standard with the footwear	1
	Moisture wicking	1	Leather/ plastazote combination	1	Diabetic lining	1	Diabetic, non-seams	1
	Kangaroo leather	1	Antibacterial lining	1	Air net antibacterial lining	1	No seems soft non-leather	1
	Diabetes-friendly, non-leather	1	Diabetes-friendly, non-leather	1	Diabetes-friendly, non-leather	1	Leather/ synthetic	1
			Soft, no seams non-leather	1	No leather, no seams	1		
			Seamless leather	1				
			Synthetic	1				
**Padding materials between the upper and lining**	Mole foam	1	As they come in prefab shoes	1	Plastazote	1	Plastazote	1
	Padded top line	1	0.2 cm foam forefoot	1	0.2 cm Topy Cellolite	1	Foam	1
	Collar and tongue	1	Plastazote	2	Latex/foam	1	Collar padding	1
	Latex	1	Collar & tongue	1	Tongue	1		
	Foam	1	Foam	1	Soft Urethane for the collar and tongue areas	2		
	Around the topline	1			Collar padding, tongue	1		
					0.3 cm	1		
**Reinforcements materials**	Stiff toe box to ensure depth	1	Anti-tear material	1	Shield tongue	1	Tape	1
	Toes	1	Tape	1	Tape	1		
	EVA	1	Counter	1	Extended M/L reinforced	1		
	The custom-made orthotic insert to offload plantar pressures	1	Strong toe cap	1	Tongue reinforcement	1		
	Buttress if required	1	Lateral buttress/bilateral		Reinforce the tongue and vamp amputated side	1		
	Anti-tear	1			1.8mm Rhenoflex heel stiffener	1		
	Non-extra	1						
	Good heel counter	1						
**Type of footwear modification**	It depends on gait & stability	2	Possibly, depending on gait		Medial (R&L)	3	M/L flares bilaterally	1
	If required	1	Lateral flare	3	Medial flares bi-laterally	1	Buttress if needed	1
	Flared outsole	1	Lateral buttress on insole component	2	Rocker: left is rigid, right is semi-rigid	1	Stretch toe box to accommodate overriding digits	1
	Toe off rocker	1	Flare	1	Rigid rocker for the left	1		
	Offloading excavation for the hallux (can be in either shoe or orthosis or both)	1	Carbon fibre stiff sole (R)	2	The device is custom-made	1		
	Stiffened sole	1	Stretch or reblock upper at left 5^th^ MPJ	1				
	Forefoot required widening to accommodate HAV	1						
**Rocker sole Apex position**	10%	1	2 cm behind the previous ulceration site (% depends on the length of his foot)	1	2 cm behind 1st MPJ R)	1	It depends on the position of the 1st and 5th MPJ – probably 3 cm behind the joint line	1
	70%	2	Proximal to MTHs, approximately 1 cm, equal 2/3 (70%) from heel to forefoot	2	Proximal to MTHs, approximately 1 cm, equal 2/3 (70%) from heel to forefoot	1	Proximal to MTHs, approximately 1 cm, equal 2/3 (70%) from heel to forefoot	1
	2 cm behind MTHs 1–5 (R&L) depends on where the patient feels stable	1	50%, and proximal to the past ulcer site	1	55-60%	3	60%	1
	Proximal to MTHs, approximately 1 cm, equal 2/3 (70%) from heel to forefoot	1	55-60%	3	1 cm proximal to MPTJ	1	55-60%	3
	I would not recommend a rocker sole on this, but these are typically my normal setup – angles of 60	1	1 cm proximal from MPJ	1	60-65%	1	52%	1
	55-60%	5	52%	2	Proximal to stump on the amputated side	1	60-70%	1
	Posterior to the met-heads	1	Based on the foot angle	1	40% or 50% on the amputated side, depending on the stump	1	Based F-scan data	1
	Relevant to the abduction of gait	1			Based on needs	1		
**Midsole materials**	EVA, softer for cushion	1	EVA shore A40-45	3	EVA shore A45	3	EVA shore A40	2
	EVA	2	EVA Shore A80	3	Mid-density EVA generic brand	1	Low-density EVA generic brand	1
	EVA Shore A80	1	EVA	3	EVA 80 shore	4	EVA	2
	EVA Shore A45	3					EVA Shore A60	1
	EVA Shore A60	1					EVA Shore A35	3
	PU or EVA Shore A35-45	1						
	EVA (Shore A ~ 40)	1						
	EVA or PU firm	1						
**Outsole materials**	High wear rubber	1	Commando Soles	1	Rubber or EVA non-slip	1	EVA non-slip	1
	EVA	1	One-piece full-length Vibram trekking flat sole Full length	1	0.6 cm cellosoft Topy	1	0.3 cm Topy cellosoft	1
	Cellosoft Topy	1	Rubber	1	Rubber carbon fibre plates	1	Rubber/EVA	1
	Rubber/ Topy	1	Grippie Vibram Tank	1	Topy Cellotop	1	Vibram Clivia	1
	Non slip Vibram Clivia	1	Vibram Clivia Non slip	1	Topy Crock	1	Light anti-slip	2
	Rubber with an anti-slip profile	1	Durable rubber for bush	1	Rubber, non-slip	2	0.4 cm rugged	1
	Cellotop (~50D shore)	1	Strong profile for bushwalking	1	0.6 cm rugged	1		
	An anti-slip, active person	1	0.6 cm rugged	1				
	0.4 cm rubber rugged	1						
**Heel materials**	EVA	1	3.5 cm	1	0.6 cm cellosoft	1	0.6 cm cellosoft	1
	Rubber or EVA depends on the activity levels of the patient	1	One-piece full-length Vibram trekking flat sole Full length	1	Rubber	1	Rubber	1
	Mid-density EVA	1	Rubber	1	Vibram Tank	1	Vibram Tank	1
	Rubber	1	Wedge	1				
	Topy Winter	1	Topy Winter	1				
	Full wedge heel preferred	1						
**Sole & Wedge materials**	EVA	1	Vibram pre-formed hiking long soles	1	EVA or rubber non-slip	3	EVA with non-slip sole	1
	Rubber or EVA depends on the activity levels of the patient	1	Rubber	1	EVA Vibram	1	Vibram	2
	EVA Vibram shore A60	2	EVA	1	Rubber	1	928 wedges, EVA	1
	Mid-density EVA generic	1	EVA wedge Shore A65 + full rubber (Vibram)	1	EVA	1		
	Rubber/Topy	1	Preferred heel sole wedge	1	Vibram	1		
	EVA, Shore A45	3	Vibram	1	Wedge, 924	1		
	Non-slip top	1	928 wedge, EVA 400	1				
	Vibram	1						

### Opening and fastening

For all four cases, the most recommended fastening method by clinicians was “Combination (Lace + Velcro)”, with 20–25 respondents consistently selecting it. Velcro fastenings also received moderate support (around 10–15 responses per case), while Laces and Zippers were far less frequently chosen, often with fewer than 10 recommendations each. The data highlight a clear clinical preference for footwear with dual fastening systems across different patient scenarios. Part of Table 3 outlines the additional suggested types of fastening and material recommendations.

### Other modifications

Footwear modification is a common recommendation for the cases, and the most common recommendation is a semi-rigid rocker sole design for Case-1 (n = 10) and Case-4 (n = 8), followed by a rigid rocker for Case-1 (n = 6) and Case-4 (n = 1). A rigid rocker sole design is also the most recommended for Case-2 (n = 7) and Case-3 (n = 9), and a buttress (n = 6) and (n = 4) for them, respectively. Re-lasting or widening (n = 4) are also recommended for Case-1 as she has HAV that needs extra room on the right foot. For Case-2 and Case-3, the extra room required is incorporated within the custom-made footwear design. Other footwear modifications include a stiffened outsole, toe-off rocker, deflection under the hallux, and re-lasting to accommodate the HAV and flared outsole. Table 3 present the details of the above footwear parameters for all the cases.

### Rocker sole design parameters

There was a common agreement on rocker sole apex position parameters and variations in apex angle parameters with greater variations in rocker angle parameters recommendations among the pedorthists. The reason for the variations is due to participants’ stability, the orientation of the metatarsal heads and the bony prominences such as region of interest to offload the plantar pressure.

Apex position recommendations for the cases vary in interpretation by the pedorthist, and the most common recommendations are at 55–60% of the length of the shoe for the cases, respectively. At 70% length, the apex position is the second most recommendations for the cases, and 1 cm proximal to the MTHs are common recommendations for all cases. Some pedorthists also recommended performing in-shoe pressure analysis to determine the apex position.

Apex angle recommendations range from 50–97 degrees, and this is mostly dependent on the alignment of the 1^st^ and 5^th^ MTHs. The most common recommendations are between 80–95 degrees for the apex angle.

Rocker angle recommendations vary between 10–30 degrees, and the most common recommendations are 10–12 degrees and 15 degrees for all cases. The participant’s balance and in-shoe pressure mapping have also been recommended to determine the rocker angle.

**Fig 5:**
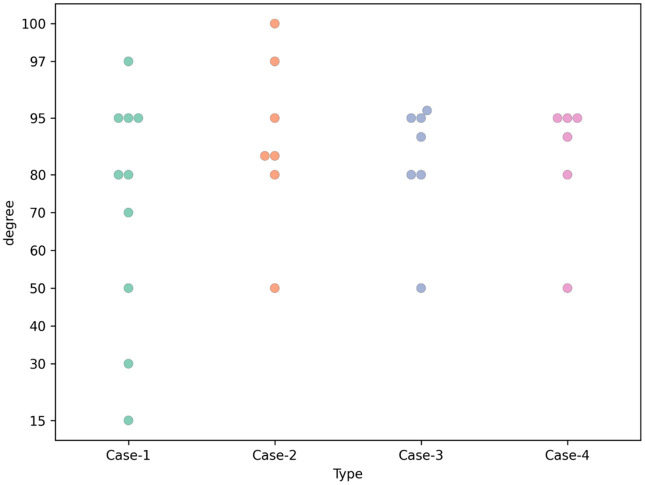
Apex angle recommended for Case-1, Case-2, Case-3 and Case-4 (scatter plot).

**Fig 6:**
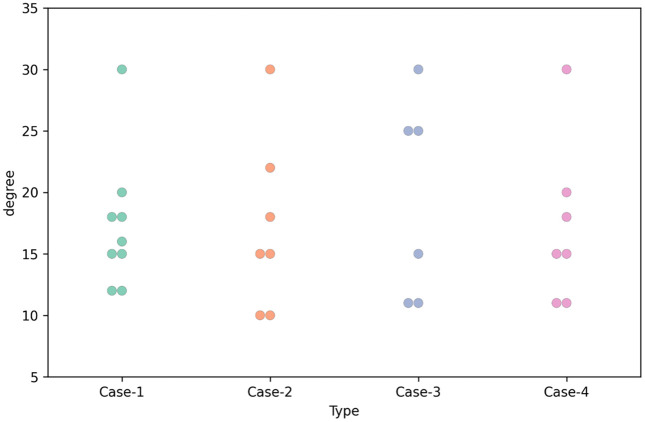
Rocker angle recommended for Case-1, Case-2, Case-3 and Case-4 (scatter plot).

The detailed descriptions and recommendations on the rocker apex position, apex angle and rocker angle are presented in Table 3 and Figs 5, 6.

### Sole materials and characteristics

There was a high level of consistency between the pedorthists around recommendations of shoe materials and characteristics for outsole, midsole and heel but there were some level of disagreement around sole and wedge materials recommendations. More variation for cases 1 and 4 than for cases 2 and 3.

EVA was the most recommended midsole material for all cases by the pedorthists, and the shore value of the recommended EVA ranged from 35 to 80. Mid-range density (35–45 Shore A) EVA is the most preferred by the pedorthists when it comes to the midsole materials selection.

Non-slip rubber is the most preferred outer sole material by the pedorthists for the cases, and hard-wearing EVA and cellosoft rubber are the other recommended outsole materials by the pedorthists. Heel materials recommendations have similar choices as the outer soles.

Sole and wedge are common recommendations for the cases by the pedorthists, and a combination of EVA wedge and non-slip rubber or Topy outer layers are the most common preferences by the pedorthists when it comes to the sole design. Table 3 also presents more detailed descriptions of the midsole, outer sole, heel, sole and wedge materials that are recommended by the pedorthists for the cases.

### Insole characteristics

There is consistency in recommendations for insole type for the cases, with some variations in case 4. There are some variations in recommending insole materials type by the pedorthist. There are more variations in recommendations for cases 1 and 4 and fewer variations in cases 2 and 3 when it comes to insole material recommendations.

### Insole type

Three different types of insoles were recommended by the pedorthists for the cases, including prefabricated insoles, custom-made insoles and the insoles that come with prefabricated medical-grade footwear. Among them, the custom-made insoles are the predominant recommendations for all the cases (n = 13, 10, 10, 8 for each case), and in some cases, this is the only recommendation. Fig 6 describes the type of insole recommendations for the cases by the pedorthists.

**Fig 7:**
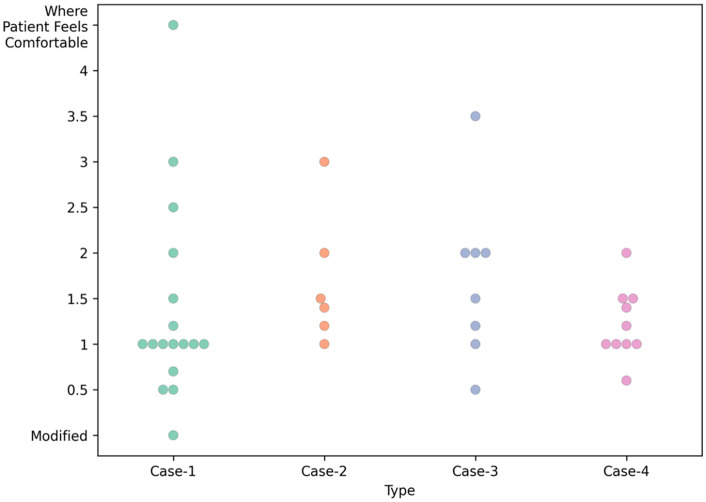
Toe spring recommended for Case-1, Case-2, Case-3 and Case-4.

### Casting method

Casting method recommendations also vary in pedorthists’ choices, and the most preferred casting methods are non-weight-bearing casting followed by semi-weight-bearing casting. In all four cases, the most frequently used casting method was non-weight-bearing, chosen by 50% of clinicians in Cases 1–3 (7, 5, and 5 clinicians respectively), and even more so in Case 4, where 66.67% (6 clinicians) selected it. The semi-weight-bearing method was also common, used by 42.86% (6 clinicians) in Case 1, 50% (5 clinicians) in Case 2, 40% (4 clinicians) in Case 3, and 33.33% (3 clinicians) in Case 4. The full-weight-bearing method was rarely applied, appearing in only Case 1 and Case 3 with 1 clinician (7.14% and 10%), and not at all in Cases 2 and 4.

### Insole materials

Insole design involves decisions on various materials used for different layers with various densities and hardness of materials based on the requirements and cases. EVA of various thicknesses and hardness is the most common recommendation for insole design as the base layer. The insole thickness recommendation is 0.5 to 1.5 cm, and the shore value is between 35–65 shore A. Table 4 presents the detailed recommendations on insole base layer material, thickness and hardness.

**Table 4:** Insole recommendations for Case-1, Case-2, Case-3, Case-4.

Parameters for insole/ Orthoses features	Case-1		Case-2		Case-3		Case-4	
	Description	n	Description	n	Description	n	Description	n
*Insole base material and thickness in cm*	EVA 1.5 cm	2	EVA 1.5 cm	2	EVA Shore A35-40	2	EVA shore A30	1
	Heat moulded Relux	1	Heat moulded Relux	1	Tri-laminate/ cork 1.5 cm	1	Tri-laminate/ cork 1.5 cm	1
	Qform 0.14 cm from heel to metatarsal heads line	1	Qform 0.14 cm from heel to metatarsal heads		High-density EVA Generic	1	Qform 0.14 cm	1
	EVA 1 cm	1	EVA shore A35-40	1	0.6 cm Thermocork X 2	1	EVA 0.5 cm	1
	EVA Cork Shore A65	1	O.3 cm	1	0.3 cm	1	EVA Cork Shore A45	1
	EVA shore A35-40	2	EVA 1 cm	1	EVA 1 cm	1	EVA shore A35	1
	EVA 3/4 length	1	EVA Cork Shore A65	1	EVA shore A45 - 50	1	3/4 length EVA	1
			EVA 3/4 length		Hard EVA 3/4 length	1	EVA 1.5 cm ground to zero at peak	1
					EVA 1.5 cm ground to zero at the apex	1		
*Insole mid-layer material and thickness*	PPT 0.3–0.6 cm	1	PPT 0.3–0.6 cm	1	EVA shore A35 and PPT (Poron)	1	EVA shore A30	1
	Q-form	1	Q-form	1	Tri-laminate PPT/Poron/ Plastazote	1	0.3 cm dual-density Urethane	1
	Mid-density EVA full-length	1	Mid-density EVA full-length	1	6mm Poron	1	Poron + layer of EVA Shore A30	1
	0.3 cm Poron blue	1	0.3 cm Poron blue	1	0.6 cm EVA 220	1	Poron/ PPT	1
	Dual-density Urethane 0.5 cm	1	0.3 cm	1	0.3 cm	1	0.5 cm Poron	1
	0.3 cm slow-release Poron	1	Dual-density Urethane 0.5 cm	1	Dual-density Urethane	1	65 celolite, 0.6 cm	1
	Shore A35 0.6 cm and Poron/ PPT 0.6 cm	1	3mm slow-release Poron	1	Slow-release Poron, EVA Shore A35	1		
	0.6 cm Poron	1	Shore 35 0.6 cm and poron/ ppt 0.6 cm	1	EVA shore A35 and PPT/ Poron	1		
	0.6 cm XRD Poron	1	0.6 cm Poron	1	0.5 cm Poron	1		
			0.6 cm XRD Poron	1	0.6 cm XRD Poron	1		
*Insole top cover material and thickness*	EVA 0.3–0.6 cm	1	EVA 0.3–0.6 cm	1	EVA shore A20, 0.3–0.6 cm	1	EVA shore A20 0.3–0.4 cm	1
	EVA 0.45 cm	1	EVA 0.45 cm	1	Tri-laminate is complete	1	Tri-laminate	1
	0.4 cm cellolite with Smooth, shiny leather full length 0.1 cm	1	0.4 cm cellolite with smooth, shinny leather full length 0.1 cm	1	Smooth, shiny leather 0.1 cm	1	0.6 cm Super soft Poron with smooth, shinny leather cover	1
	0.6 cm Plastazote	1	0.6 cm Plastazote	1	0.6 cm Plastazote	1	EVA Perf A35 0.175 cm thickness	1
	Plastazote	1	0.2 cm	1	0.2 cm	1	0.2 cm, EVA Shore A20	1
	0.3 cm Luna lastic, Shore A25	1	Plastazote	1	EVA Marilon Perf	1	0.3 cm shore A20 EVA	1
	0.4 cm EVA shore A20	1	0.3 cm Luna lastic, Shore A25	1	EVA Shore A22	1	0.3 cm Plastazote	1
		1	0.4 cm EVA shore 20	1	0.4 cm EVA shore 20	1	0.2 cm Plastazote	1
	0.2 cm Plastazote	1	0.3 cm Plastazote	1	0.5 cm Plastazote	1		
			0.2 cm Plastazote	1	0.2 cm Plastazote	1		
*Additional information on insole materials*	Full-length carbon plate for right with a prostheses toe spacer for missing hallux	1	Possibly cork inlays	1	Sanded thin at distal toes	1	Low-density EVA base to match the insole shape – no thickness	1
	0.6 cm EVA Shore A45 as middle layer lateral buttress, 1 cm support proximal cuboid by caving into the mould, 12 cm high buttress on lateral side bi-lateral	1	Full-length carbon plate for right with a prostheses toe spacer for missing hallux	1	Prosthetic element EVA shore A35	1	Dr Comfort gel insole with mods	1
			0.6 cm EVA Shore A45 as middle layer lateral buttress, 1 cm support proximal cuboid by caving into the mould, 12 cm high buttress on lateral side bi-lateral	1			Add to the bottom of the provided insole soft EVA and sand ding to grind out high-pressure points	1
							Soft prosthetic element shore A15 or less	1
*Additional arch support*	Slow-release PPT 0.6 cm	1	Possibly	1	As the cast was taken, it should not need additional arch support	1	EVA shore A30, 1 cm	1
	Cast from the foot, it should not need it	1	1 cm	1	as required	1	6 mm taken out of the mould	1
	As required	1	0.5 cm EVA	1	0.5 cm	1	0.6 cm EVA shore A35	1
	0.2 cm EVA	1	0.5 cm	1				
	0.6 cm	1						
	plus 1 cm	1						
*Metatarsal dome*	Pre-1st and 5th MPJ – PPT – 0.6 cm	1	PPT, behind 3rd MTH (L), 0.6 cm	1	Rt 1st MPJ. 0.6 cm PPT	1	0.6 cm PPT slow-release (Poron)	1
	0.4 cm proximal from MTH	1	220 EVA 0.6 cm distal 3rd MTH left	1	0.6 cm Poron MD proximal to amputation site on L	1	Right pre met 0.6 cm Poron.	1
	Poron met done 0.6 cm	1	Poron Metdome	1			0.6 cm Poron MD R proximal 2nd MTH	1
	0.4 cm, proximal MTHs	1	0.5 cm met dome; proximal MTHs	1			0.4 cm taken out of the mould	1
	plus 0.8 cm	1	1 cm	1			0.6 cm Poron	1
	0.5 cm Poron	1	0.3-0.4 cm	1			0.3-0.4 cm	1
*Metatarsal bar*	EVA 0.3–0.6 cm behind metatarsal heads	1	EVA behind the metatarsal heads, min 0.6 cm	1			Possibly	1
	Proximal 1 cm approx., from the angle of the 1^st^ to the 5^th^	1	EVA, depending on how he feels about offloading	1			Left 0.6 cm Poron pre MTHs #1–5	1
	0.8 cm	1	Pre # 3, #1, #5 met bilateral Mid density EVA 0.8 cm	1			0.6 cm Poron MB proximal L 4^th^-5^th^	1
	0.4 cm, prox MTHs	1	220 EVA 0.6 cm + proximal 1^st^-5^th^ met heads	1				
	0.5 cm EVA Shore A35	1	0.4 cm proximal from MTHs	1				
			0.8 cm met bar; prox MTHs	1				
*Metatarsal pad*	As per In-shoe pressure mapping	1	Not recommend-ed	1	0.3 cm Poron blue proximal to 1^st^ met head R/F	1	Not recommend-ed	1
	0.3 cm urethane	1			0.3 cm	1		
	0.3-0.4 cm				0.3-0.4 cm	1		
*Local cushioning by removal of materials and adding cushion*	Excavation in areas where high plantar pressure is present	1	Excavation under previous ulceration site	1	Plantar aspect and stump face – 0.6 cm Super soft Poron	1	Remove hard materials and replace them with slow-release Poron, under the boney proms bilaterally	1
	Qform to have a shape dropout at the right hallux	1	Left #3 MPJ, right #1 MPJ	1	Removal of approx. 0.3 cm under 1st R met and replaced with 0.3 cm Poron blue	1	SLR Poron at bony prominence	1
	Under 1st and 5th Urethane 0.3 cm	1	Slow release Urethane 3 mm	1	Additional Poron	1	SLR Poron	1
	Offload 1st + 5th MTP, by adding to mould	1	0.5 cm offload under 5^th^, 3rd MPJs by adding to the mould on the left side	1	0.6 cm SR Poron	1		
	Slow-release Poron 0.3–0.6 cm in the area of the closed ulcer	1	0.6 cm slow-release Poron	1				
	At ulcer site slow-release Poron 0.6 cm	1						
*Additional information on insole design/modification features*	Excavation for hallux where ulcer has occurred	1	Prostheses toe spacer for missing R hallux	1	Medial buttress (R&L)	1	Toe filler poron/ plastazote	1
	Trial & test pressure	1	The depths and density of materials used will depend on supporting feet and controlling function	1	Left is a partial foot orthosis wrapping over the stump up to the dorsal midfoot – with a carbon plate. Right, no carbon plate	1	small prosthesis spacer fitted to the left for missing digits.	1
	Right carbon plate or Qform to be full length (reduce, to reduce hallux plantar pressure in gait.	1			Carbon fibre plate	1	Mods based on F-scan	1
	A custom-made foot orthosis.	1			Prosthesis element on the amputated side	1		
	Reassessing high-pressure points	1			Prosthetic element, with the anterior shield to take the load away from the stump on the amputated site	1		
	In-shoe pressure mapping to measure the effectiveness of the offloading (all is relative)	1			Toe filler left	1		
					All modifications based on F-scan findings	1		

Medium-density EVA (Shore 30-35A), PPT or Poron, Qform are the commonly recommended mid-layer materials for insole design. The recommended thickness of PPT or Poron is between 0.3–0.6 cm, and other recommended materials are XRD Poron, cellolite and dual-density Urethane. Table 4 presents a detailed description of mid-layer materials for the insole design for the cases.

A number of materials were recommended, such as insole top covers by pedorthists. The most commonly used top covers were 0.3–0.6 cm thick, softer density EVA (Shore 20A) and 0.2–0.6 cm thick Plastazote. Supersoft Poron and smooth, shiny leather were recommended by some pedorthists. The insole top cover materials recommendations are described in Table 4 presents any additional information provided by the pedorthists for the insole design.

Additional arch support to increase the contact area and to offload was recommended by some pedorthists. Common materials for the additional arch support were Slow-release Poron, and medium-density EVA. The recommended thickness was between 0.5–1 cm. 4 outlines the details for each case.

Pedorthists commonly recommend a metatarsal dome between 0.3–0.6 cm thick, with 0.6 cm thickness being the predominantly recommended thickness, and the suggested materials are Poron or PPT. The positioning of the metatarsal dome was recommended proximal to the MTHs. Table 4 provides more information on the recommended metatarsal dome by the pedorthists.

Pedorthists also recommended metatarsal bars for the cases, and common materials for the bars are EVA with 0.3–0.8 cm thickness and medium-density Poron with 0.6 cm thickness. More information is provided in Table 4.

Pedorthists also recommended a metatarsal pad when indicated by the condition for the cases, and commonly preferred materials are Poron with 0.3–0.4 cm thickness. More information is provided in Table 4. The pedorthists have provided a number of additional information on insole design and modification, and the details are presented in Table 4.

### Addressing adherence

Common challenges related to adherence described by the pedorthists in the survey were the appearance of the footwear, weight and profile, insole material and thickness that determine the depth of the footwear and adapting to shoe and insole modifications. Pedorthists reportedly apply and recommend various techniques to overcome adherence-related challenges in their practice.

**Fig 8:**
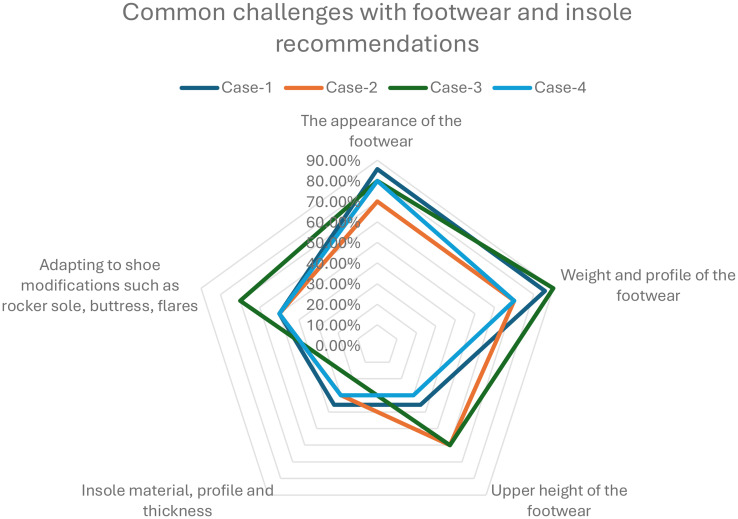
Common Challenges with Footwear and Insole Recommendations for Case-1, Case-2, Case-3 and Case-4 (Spider Web Graph).

**Fig 9:**
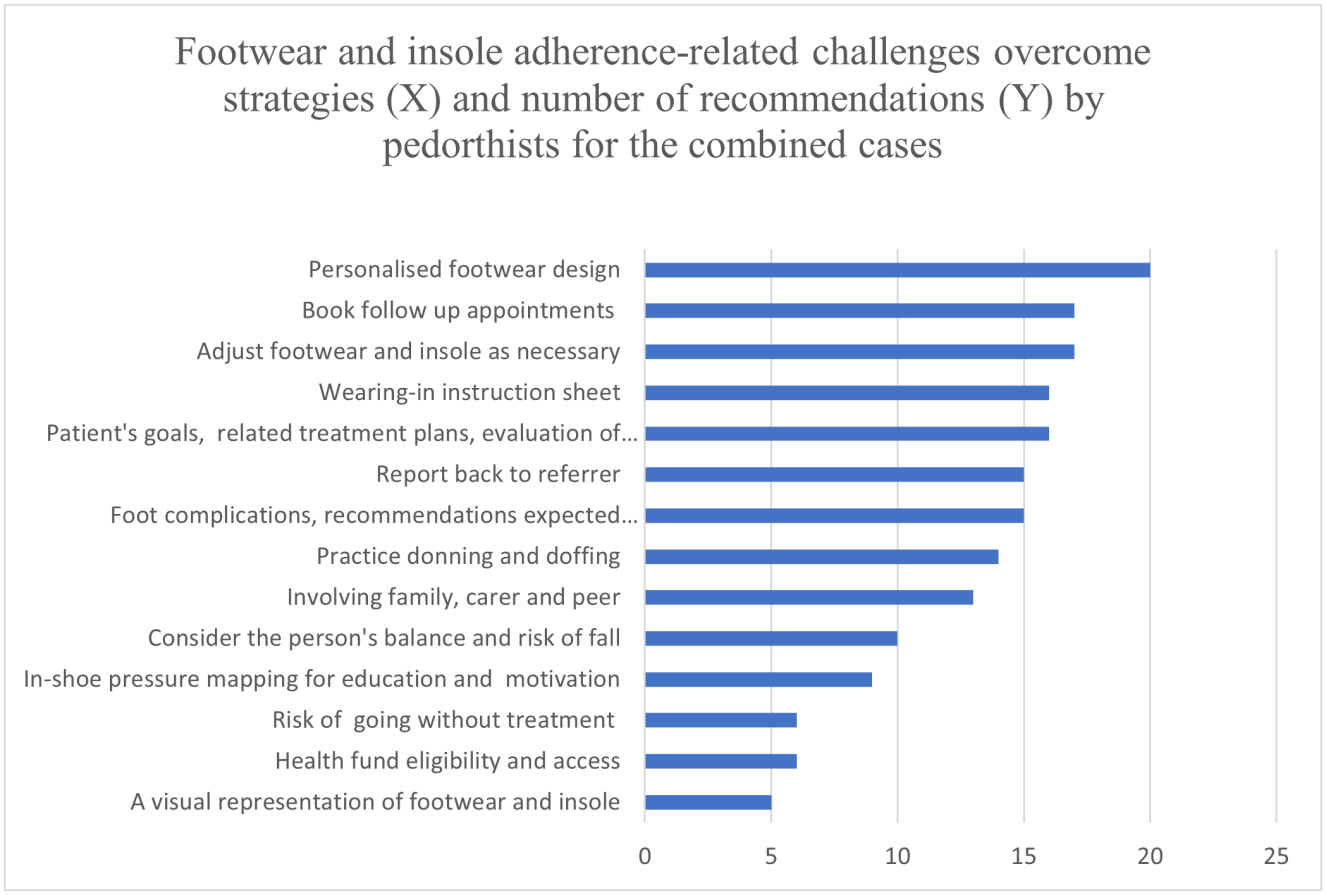
Footwear and insole adherence-related challenges overcome strategies by pedorthists for the combined case.

Some common strategies are designing person-centric footwear, explaining the benefit of wearing the footwear that can reduce the risk of further complications, and footwear designed for specific intended use such as outdoor and indoor use, bushwalking and such. Cultural and gender-specific footwear design, intended use of the footwear, and engaging the client and family or friends in design-related decision-making with evidence from clinical assessment and plantar pressure-related data are some key techniques that the pedorthists apply, as reported by some pedorthists in the open text sections. A suitable fastening system, easy donning and doffing, instructions on the wearing-in process, review appointments to monitor adherence and outcome, necessary adjustments and referring back to referring podiatrist or multidisciplinary team and advising on suitable fund options are also some approaches that pedorthists apply to increase adherence of the clients. More detailed information on pedorthists’ common practice to overcome techniques are presented in Figs 7–9. The total number of pedorthists who responded to Cases 1, 2, 3 and 4 are n = 19, 11, 10 and 10.

### Evaluating the plantar pressure offloading success

Pedorthists use three different approaches to evaluate the offloading success of the prescribed devices: clinical judgements based on experience, recurrence of ulcers and in-shoe pressure mapping and analysis. In-shoe pressure analysis is the most used method to evaluate pressure offloading success (n = 11, 8, 8, 8), followed by clinical judgment (n = 5, 6, 6, 5) and ulcer recurrence (n = 5, 4, 3, 4) for each case.

Across all four clinical cases, the most used strategy for footwear evaluation was “Clinical judgement”, consistently receiving around 9–11 clinician responses per case. In-shoe pressure analysis was used less frequently, with approximately 5–7 respondents, while “Ulcer recurrence” as a feedback method was the least employed, generally chosen by fewer than 5 clinicians per case. This reflects a strong reliance on subjective clinical assessment rather than objective pressure data or recurrence tracking in footwear evaluation practices.

## Discussion

This section discusses the prescribing patterns of Australian pedorthists across four standardized patient scenarios of increasing biomechanical complexity, all at risk for diabetic neuropathic forefoot ulceration. To orient the reader, the discussion is structured in two main parts. First, each case is examined individually to explore patterns of agreement or variation in prescriptions and the influence of case complexity and practitioner qualifications. Then, broader thematic insights across all cases are discussed, including consistency in certain footwear or insole parameters, alignment with best practice guidelines, and implications for future standardization. Finally, we reflect on limitations and contextual factors influencing variability in prescribing practices.

### Complexity level and general assessment of the cases

***Case-1:*** The biomechanical factors, in this case, are simpler than subsequent cases, requiring less skill and technical knowledge to address; *h*ence, the response rate is highest for this case, and the recommendations in footwear type, upper height, heel height, and toe spring have the greatest consensus compared with other cases.

***Case-2:*** This case is relatively complex in biomechanical aspects, and pedorthists with the qualifications of C Ped and C Ped CM were able to handle this case. Variations in footwear type recommendation are observed here, and that continues for other features such as upper height, heel height, rocker sole profile and insole design characteristics. The intended activity and lifestyle of the participant would influence the recommendations.

***Case-3:*** This case is relatively complex in biomechanical nature, and pedorthists with the qualifications of C Ped and C Ped CM were able to handle this case. Case-3 also shows similar variations patterns to Case-2 except for the common consensus on custom-made footwear but variations in other footwear and insole parameters.

***Case-4:*** This case is relatively complex biomechanically, and pedorthists with the qualifications of C Ped CM were able to handle this case. The main reasons are the over-riding digits that often require fully custom-made orthopedic boots, and only the C Ped CMs have the skill set to handle the case. The possible variations in footwear type recommendations also may vary due to age and gender-specific preferences on aesthetics, and custom-made footwear options could have been considered a barrier to adherence as the participant was a female.

This study has shown that for the most biomechanically simple patient (Case-1), there was a reasonable amount of consistency in prescribing footwear type, upper height, rocker profile, insole type and other insole design characteristics. The heel height and toe spring recommendations vary between respondents for Case-1. This could be what brand and style of footwear they could offer from the prefabricated medical grade footwear with modification, and the rocker sole modification would also re-define heel height based on individual pedorthist’s and patient’s preferences, in-shoe pressure analysis data and balance issues.

For more complex cases such as (Cases 2, 3 and 4), there was a great deal of variation in prescribing approaches. This may be due to the different skill levels of the pedorthists to handle those cases and scope of practice, [24] available options of footwear type supply in their practices and variation in material supply for manufacture and modifying insoles, health fund availability, patient’s preferences and intended activity. In Australia, pedorthists registered with APRB with three different skill levels [24].

The survey allowed the participants to answer Case-related questions that were comfortable for them to answer and relevant to their regular scope of practice. Best practice [17,18] would suggest that pedorthists recommend prefabricated medical grade footwear with modifications for the less complex cases such as Case-1 and custom-made footwear for the more complex cases such as Case-2 to Case-4. Similarly, based on existing practice guidelines [17,27], participants were expected to recommend custom-made insoles (foot orthotics) for all Cases [17,18].

Ideally, in-shoe pressure mapping systems would be used to evaluate offloading success rather than clinical judgement and waiting on recurring ulcers [17,18]. However, variations were anticipated due to the different skill levels of the participating pedorthists, the scope of practice and available clinical and device-specific resources, the patient’s preferences and suitability with the intended use, and available funds for therapy.

These results show limited consensus in prescribing practices, particularly as cases become more complex, although some features could be standardised in prescribing footwear [28]. For example, a person with diabetes, neuropathy and a regular foot structure and no to minor foot deformity could be prescribed prefabricated medical grade footwear and additional modification for increased deformity level, and fully custom-made footwear for a complex foot structure and deformation. This approach is within the guidelines of DFA guidelines [17] recommendations. A custom-made insole can be commonly recommended for a person with diabetes, neuropathy, and any foot complications. One of the problems is that there are a lot of variations in prescribing practices but little evidence to support one approach over another. This research has explored the pathways to tighten up the variations in an evidence-based way.

All these variations in prescribing patterns, such as footwear and insole type, design and modification features, could be explored and standardised more for each case through a person-centric design approach. Hence, the variations were tested in a series of Trials to have a precise prescription for each individual for the foot pathology, comorbidity, intended activity, mobility status and personal preferences.

Overall, the purpose of this study was to identify variations in prescribing practices. Table 5 shows the extent of variations in consensus by Australian pedorthists in prescribing footwear and insole design and modification when they see various typical cases.

**Table 5:** Summary of consensus on footwear and insole design and modification prescribing by Australian pedorthists.

Footwear and insole design and modification features		Agreements on recommendations by the pedorthists			
		Case-1	Case-2	Case-3	Case-4
Footwear type	Custom-made pedorthic footwear				
	Prefabricated pedorthic footwear				
	Prefabricated pedorthic footwear with modification				
Footwear upper height	Low cut				
	High cut/Bottine				
	Extra high cut				
Heel counter	Standard				
	Extended Med/Lat				
Heel height	1-2 cm				
	2.1-3 cm				
	3.1-3.5 cm				
Toe spring	0.5-1 cm				
	1.1-1.5 cm				
	1.6-2 cm				
	2.1-3 cm				
	3.1-3.5 cm				
Rocker profile	No rocker				
	Apex position at 50–60%				
	Apex position at 61–70%				
	Apex angle 80°-94°				
	Apex angle 95°-97°				
	Rocker angle 10°-15°				
	Rocker angle 16°-20°				
	Rocker angle 21°-25°				
Insole type	Prefabricated				
	Custom-made				
Casting method	Non-weight-bearing				
	Semi-weight-bearing				
	Full-weight-bearing				
Metatarsal additions	MLA increase 0.1–0.5 cm				
	MLA increase 0.6–1 cm				
	Metatarsal addition 0.5–0.8 cm				
	Location 0.4–1 cm prox. to MTHs				
Insole modification	Removal of hard material				
	Local cushioning				
	Replacement of top cover				
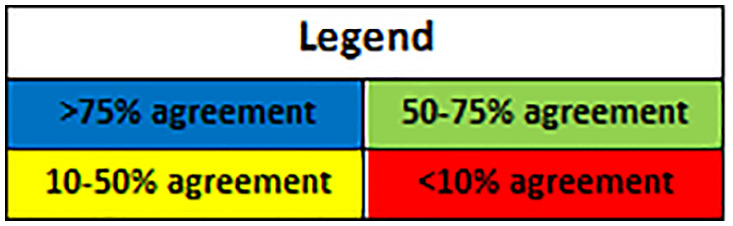					

### Footwear type

Whether it is custom-made or a prefabricated medical grade (Pedorthic footwear) footwear with or without modifications to be recommended, it is guided by foot pathology, foot structure, comorbidity and patient preferences. The variations are within the evidence in the literature, [17,18] and pedorthists follow the best practice statement as seen in the case responses.

### Footwear upper height

There is a common practice of recommending higher upper for reducing plantar forefoot pressure, although there is a lack of scientific evidence for the influence of higher upper vs. low-cut shoes in the efficacy of plantar pressure reduction [18] but the higher upper may reduce shear forces inside the shoe at the forefoot by increasing contact area around the ankle [29]. The upper height recommendation is based on foot pathology, comorbidity and mostly on the patient’s preferences. The evidence in the literature is weak for upper recommendations [18], but pedorthists tend to make recommendations for ankle-high or even higher upper design for the complex foot when the patient agrees, and this approach is supported by the best practice statement [17]. For low complexity and when there is no pathological indication for a higher-cut upper design, pedorthists commonly recommend a low-cut shoe, as seen in case 1.

### Heel counter

Heel counter design is predominantly decided by the pedorthists based on foot pathology, and it is less influenced by the patient’s choice as it is invisible, does not affect the appearance of the footwear, and the patient is more motivated by the comfort and ease of walking [17].

### Heel height and toe spring

Evidence for the above two design parameters is very limited in the literature [18]. However, these are very important parameters for footwear design and influence the pressure offloading capacity and balance of the patient [28]. For the prefabricated medical-grade footwear range, the heel height and the toe spring are guided by the footwear manufacturer’s specification on the shoe last, and pedorthists sometimes modify them as per the foot and lower limb assessment and in-shoe plantar pressure data, also assessing the balance of the patient. Pedorthists use foot assessment outcomes, patient preferences, and balance to determine heel height and the toe spring as reported for the relevant cases. Hence, the recommendations on the heel height and toe spring represent such a variation.

### Rocker profile

Apex position and apex angle are guided by the orientations of the MTHs [18,28]. The variations in recommendations are generally due to offloading requirements, the target MTH, the patient’s balance [28] and aesthetics requirements [18]. The same factors also guide rocker angle recommendations, and the recommendations are within the range recommended in the best practice statements [17,18].

### Insole type

Custom-made insoles are the most common recommendation by the pedorthists for all cases with the least variations, and it is consistent with the evidence [18].

### Casting method

The variations in the casting method for insole design are within two types of casting methods, and it is also within the range of variations in the literature [18]. However, recent evidence recommends non-weight-bearing casting methods and digital optimisation of the cast for increased contact area and optimum offloading [30].

### Insole design characteristics

Variations in recommendation for these features are observed among the practitioners and the variations are for the metatarsal additions in the form of a metatarsal pad, bar or dome and their position thickness and materials [18]. These features are also guided by the anatomical, biomechanical [28] and patients’ feedback on comfortability and preferences [18].

### Comparison with International practices

While Australian pedorthists aim to align with global principles for offloading footwear prescription, this study identified significant variability in specific design choices across the country. The most common differences were seen in heel height, toe spring, and material selection. Such variability suggests that footwear prescription in Australia is more individualised and flexible. In contrast, countries like those in North America tend to follow stricter and more uniform guidelines due to centralised healthcare policies, insurance requirements, and standardised training [31,32]. International frameworks, such as those provided by the IWGDF, promote consistency in prescription, supporting more predictable clinical outcomes [33–35]. A lack of standardisation may contribute to variation in patient adherence, effectiveness of pressure offloading, and the risk of ulcer recurrence or delayed healing [36–38].

### Limitations of this study

This study has several limitations, primarily due to its survey-based design. Although the response rate was reasonable given the small size of the pedorthic workforce in Australia, fewer than half of all registered pedorthists participated. This limits the generalizability of the findings—not only to international contexts, but even within the Australian pedorthic community. Moreover, it is possible that those who chose to participate were more confident in their clinical approach or more aligned with best-practice recommendations, which may have led to an overestimation of the consensus and adherence to evidence-based prescribing.

The use of standardized, hypothetical case scenarios enabled controlled comparison across respondents but may not fully reflect the complexities of real-world clinical decision-making. Factors such as face-to-face interaction, psychosocial considerations, and resource availability were inherently limited in this format. While the cases were informed by audit data [25] and reviewed by clinicians to improve realism, the absence of person-centred nuances likely influenced some prescribing decisions.

The reliance on self-reported data introduces potential for recall bias and social desirability bias, particularly in reflecting on typical prescribing practices. Additionally, because the study used a single data source (survey), triangulation with other data types such as clinical audits, interviews, or observational studies was not possible. Incorporating such methods in future research would strengthen validity and contextual depth.

Lastly, the findings are grounded in the Australian healthcare and training context. Variations in funding structures, regulatory frameworks, and professional roles may limit the applicability of results to other countries.

## Conclusions

Based on these survey results, there is a high level of variation and little consensus around the best way to treat patients with diabetes and neuropathy for forefoot plantar ulceration prevention. Some of this variation is warranted due to the different skill levels of the pedorthists and patients’ treatment goals, health fund availability and adherence-related matters, which further justifies the need for this study.

Some standard parameters for footwear design for some patients include prefabricated footwear with or without modification for low to moderate complexity and custom-made footwear for higher complexity cases. Custom-made insoles should be recommended for all levels of complexity for people with diabetes and neuropathy [18,27]. The design and modification features of all these devices need to be tailored for individuals based on their pathology, comorbidity, mobility status, intended activity and lifestyle.

No one size fits all, but with an effective sample size and person-centric study design, there is the potential to standardise this further [39].

## Supporting information

S1 Table Evidence summary. Summary of evidences on footwear and insole design and modification prescribing by Australian pedorthists.
(DOCX)

S1 File Appendices. Survey questionnaire and the hypothetical cases.
(DOCX)

## Abbreviations

APRB: Australian Pedorthists Registration Board
PAA: Pedorthic Association of Australia

## References

[1] Boulton AJM, Vileikyte L, Ragnarson-Tennvall G, Apelqvist J. The global burden of diabetic foot disease. Lancet. 2005;366(9498):1719–24. doi: 10.1016/S0140-6736(05)67698-2 16291066

[2] Waaijman R, de Haart M, Arts MLJ, Wever D, Verlouw AJWE, Nollet F, et al. Risk factors for plantar foot ulcer recurrence in neuropathic diabetic patients. Diabetes Care. 2014;37(6):1697–705. doi: 10.2337/dc13-2470 24705610

[3] Ghanassia E, Villon L, Thuan Dit Dieudonné J-F, Boegner C, Avignon A, Sultan A. Long-term outcome and disability of diabetic patients hospitalized for diabetic foot ulcers: a 6.5-year follow-up study. Diabetes Care. 2008;31(7):1288–92. doi: 10.2337/dc07-2145 18390801 PMC2453665

[4] Peters EJG, Armstrong DG, Lavery LA. Risk factors for recurrent diabetic foot ulcers: site matters. Diabetes Care. 2007;30(8):2077–9. doi: 10.2337/dc07-0445 17507693

[5] Pound N, Chipchase S, Treece K, Game F, Jeffcoate W. Ulcer-free survival following management of foot ulcers in diabetes. Diabet Med. 2005;22(10):1306–9. doi: 10.1111/j.1464-5491.2005.01640.x 16176187

[6] Molines-Barroso RJ, Lázaro-Martínez JL, Aragón-Sánchez J, García-Morales E, Beneit-Montesinos JV, Álvaro-Afonso FJ. Analysis of transfer lesions in patients who underwent surgery for diabetic foot ulcers located on the plantar aspect of the metatarsal heads. Diabet Med. 2013;30(8):973–6. doi: 10.1111/dme.12202 23600614

[7] Armstrong DG, Boulton AJM, Bus SA. Diabetic Foot Ulcers and Their Recurrence. N Engl J Med. 2017;376(24):2367–75. doi: 10.1056/NEJMra1615439 28614678

[8] Levin ME, O’Neal L. The diabetic foot: pathophysiology, evaluation and treatment. 1988.

[9] Cavanagh PR, Boulton AJM, Sheehan P, Ulbrecht JS, Caputo GM, Armstrong DG. Therapeutic footwear in patients with diabetes. JAMA. 2002;288(10):1231; author reply 1232-3. doi: 10.1001/jama.288.10.1231 12215125

[10] Reiber GE, Vileikyte L, Boyko EJ, del Aguila M, Smith DG, Lavery LA, et al. Causal pathways for incident lower-extremity ulcers in patients with diabetes from two settings. Diabetes Care. 1999;22(1):157–62. doi: 10.2337/diacare.22.1.157 10333919

[11] Chapman JD. Improving the design of the curved rocker shoe for people with diabetes. University of Salford. 2014.

[12] Lázaro-Martínez JL, Aragón-Sánchez J, Alvaro-Afonso FJ, García-Morales E, García-Álvarez Y, Molines-Barroso RJ. The best way to reduce reulcerations: if you understand biomechanics of the diabetic foot, you can do it. Int J Low Extrem Wounds. 2014;13(4):294–319. doi: 10.1177/1534734614549417 25256280

[13] Waaijman R, Arts MLJ, Haspels R, Busch-Westbroek TE, Nollet F, Bus SA. Pressure-reduction and preservation in custom-made footwear of patients with diabetes and a history of plantar ulceration. Diabet Med. 2012;29(12):1542–9. doi: 10.1111/j.1464-5491.2012.03700.x 22540919

[14] Cavanagh PR, Ulbrecht JS. Clinical plantar pressure measurement in diabetes: rationale and methodology. The Foot. 1994;4(3):123–35. doi: 10.1016/0958-2592(94)90017-5

[15] Bus S, Armstrong D, Van Deursen R, Lewis JE, Caravaggi C, Cavanagh P, et al. IWGDF guidance on footwear and offloading interventions to prevent and heal foot ulcers in patients with diabetes. Diabetes/Metabolism Research and Reviews. 2016;32:25–36. 26813614 10.1002/dmrr.2697

[16] Healy A, Naemi R, Chockalingam N. The effectiveness of footwear as an intervention to prevent or to reduce biomechanical risk factors associated with diabetic foot ulceration: a systematic review. J Diabetes Complications. 2013;27(4):391–400. doi: 10.1016/j.jdiacomp.2013.03.001 23643441

[17] van Netten JJ, Lazzarini PA, Armstrong DG, Bus SA, Fitridge R, Harding K, et al. Diabetic Foot Australia guideline on footwear for people with diabetes. J Foot Ankle Res. 2018;11:2. doi: 10.1186/s13047-017-0244-z 29371890 PMC5769299

[18] Ahmed S, Barwick A, Butterworth P, Nancarrow S. Footwear and insole design features that reduce neuropathic plantar forefoot ulcer risk in people with diabetes: a systematic literature review. J Foot Ankle Res. 2020;13(1):30. doi: 10.1186/s13047-020-00400-4 32498719 PMC7271493

[19] Bus SA. Foot structure and footwear prescription in diabetes mellitus. Diabetes Metab Res Rev. 2008;24 Suppl 1:S90-5. doi: 10.1002/dmrr.840 18386782

[20] NSW EH. Footwear and orthotics. 2021. https://www.enable.health.nsw.gov.au/prescribers/forms/footwear_and_orthotics

[21] Centres NAoD. Nadc collaborative interdisciplinary diabetes high-risk foot service standards 2018. 2018. https://nadc.net.au/national-standards/

[22] Cavanagh PR, Bus SA. Off-loading the diabetic foot for ulcer prevention and healing. J Am Podiatr Med Assoc. 2010;100(5):360–8. doi: 10.7547/1000360 20847350

[23] Board APR. Pedorthists register 2019. 2019. https://www.aprb.org.au/content.aspx?page_id=78&club_id=865769

[24] Australia PAo. Find a certified pedorthist. 2019. https://pedorthics.org.au/find-a-certified-pedorthists/

[25] Ahmed S, Barwick A, Sharma A, Hasan MZ, Kabir MA, Nancarrow S. Examining characteristics of those who receive pedorthic services: A clinical audit. PloS one. 2024;19(7):e0304443. 10.1371/journal.pone.0304443 PMC11216586 38950041

[26] Qualtrics. https://www.qualtrics.com/research-suite/

[27] Kaminski M, Golledge J, Lasschuit J, Heinz-Schott K, Charles J, Cheney J. Australian guideline on prevention of foot ulceration: Part of the 2021 Australian evidence-based guidelines for diabetes-related foot disease; version 1.0. Brisbane: Diabetes Feet Australia, Australian Diabetes Society. 2021. 10.1186/s13047-022-00534-7 PMC9258081 35791023

[28] Bus SA, Zwaferink JB, Dahmen R, Busch-Westbroek T. State of the art design protocol for custom made footwear for people with diabetes and peripheral neuropathy. Diabetes Metab Res Rev. 2020;36 Suppl 1(Suppl 1):e3237. doi: 10.1002/dmrr.3237 31845547 PMC7154634

[29] Praet SFE, Louwerens J-WK. The influence of shoe design on plantar pressures in neuropathic feet. Diabetes Care. 2003;26(2):441–5. doi: 10.2337/diacare.26.2.441 12547877

[30] Telfer S, Woodburn J, Collier A, Cavanagh PR. Virtually optimized insoles for offloading the diabetic foot: A randomized crossover study. J Biomech. 2017;60:157–61. doi: 10.1016/j.jbiomech.2017.06.028 28687150

[31] Care D. Standards of care in diabetes—2023. Diabetes care. 2023;46:S1–S267. 10.2337/dc23-Sint PMC9810461 36507647

[32] Collings R, Freeman J, Latour JM, Paton J. Footwear and insole design features for offloading the diabetic at risk foot-A systematic review and meta-analyses. Endocrinol Diabetes Metab. 2020;4(1):e00132. doi: 10.1002/edm2.132 33532602 PMC7831212

[33] Schaper NC, van Netten JJ, Apelqvist J, Bus SA, Fitridge R, Game F, et al. Practical guidelines on the prevention and management of diabetes-related foot disease (IWGDF 2023 update). Diabetes Metab Res Rev. 2024;40(3):e3657. doi: 10.1002/dmrr.3657 37243927

[34] Colò G, Fusini F, Melato M, De Tullio V, Logrieco G, Leigheb M, et al. The effectiveness of shoe modifications and foot orthoses in conservative treatment of lesser toe deformities: a review of literature. Musculoskelet Surg. 2025;109(3):225–32. doi: 10.1007/s12306-024-00871-9 39500821

[35] Jannah F, Sriyono S, Armini NKA, Suraya AS. Effectiveness of footwear and insole design to prevent risk foot ulcer in people with diabetes: A systematic review. Indones Nurs J Educ Clin. 2025;19(2):109–21.

[36] Bus SA, van Deursen RW, Armstrong DG, Lewis JEA, Caravaggi CF, Cavanagh PR, et al. Footwear and offloading interventions to prevent and heal foot ulcers and reduce plantar pressure in patients with diabetes: a systematic review. Diabetes Metab Res Rev. 2016;32 Suppl 1:99–118. doi: 10.1002/dmrr.2702 26342178

[37] Maciejewski ML, Reiber GE, Smith DG, Wallace C, Hayes S, Boyko EJ. Effectiveness of diabetic therapeutic footwear in preventing reulceration. Diabetes Care. 2004;27(7):1774–82. doi: 10.2337/diacare.27.7.1774 15220265

[38] Chirilă A, Avădanei M-L, Mihai A, Costea M, Iovan-Dragomir A, Seul A. Advances in diabetic footwear and plantar pressure distribution devices: literature review on design, efficacy, and patient outcomes. Industria Textila. 2025;76(01):107–18. doi: 10.35530/it.076.01.2024149

[39] Catalfamo P, Moser D, Ghoussayni S, Ewins D. Detection of gait events using an F-Scan in-shoe pressure measurement system. Gait Posture. 2008;28(3):420–6. doi: 10.1016/j.gaitpost.2008.01.019 18468441

