# Supplementary material for: Current practice in prescribing footwear and insoles to reduce the risk of neuropathic plantar forefoot ulceration and re-ulceration in people with diabetes
Source: PLoS One. 2026 Feb 10;21(2):e0341594. doi: 10.1371/journal.pone.0341594 (PMC12890116; doi:10.1371/journal.pone.0341594)
Supplement: S1 Table — Summary of evidences on footwear and insole design and modification prescribing by Australian pedorthists. (DOCX) [file pone.0341594.s001.docx]

| Intervention | Treatment goal | Strength of evidence | Current pedorthic practice |
| --- | --- | --- | --- |
| Footwear type and upper height | Protect the foot  Accommodation for specific activity  Aesthetics  Plantar pressure reduction | Good evidence for footwear type recommendations  Limited evidence for upper height  Patient preference plays an important part  Cost is an important determinant | Wide variations in practice based on upper type, upper height and upper material. |
| Heel height | Reduce forefoot plantar pressure  Increase propulsion  Minimize risk of falling  Aesthetics | Good evidence for plantar pressure offloading efficacy and influence on balance | Variations in practice according to pedorthists’ choices on heel height selection for individual cases.  There is sufficient evidence to recommend heel height between 1-2 cm for optimum forefoot plantar pressure offloading |
| Toe Spring | Reduce forefoot plantar pressure  Increase propulsion  Minimize risk of falling  Aesthetics | Good evidence for plantar pressure offloading efficacy and influence on balance | Variations in practice according to pedorthists’ choices on toe spring height selection for individual cases. Common agreement on 0.5-1.5 cm toe spring for the individual cases |
| Rocker Profile | Reduce forefoot plantar pressure  Increase propulsion  Minimize risk of falling  Aesthetics | Strong evidence for plantar pressure offloading efficacy and influence on balance | Variations in practice according to pedorthists’ choices on rocker apex position and rocker angle selection for individual cases. Common agreement on apex position at 50-60% length of the shoe, rocker apex angle between 95°-97°, rocker angle 10°-15° for the individual cases. |
| Insole type and insole casting methods | Increase base of contact and cushion under the foot  Reduce forefoot plantar pressure  Reduce mechanical pressure and stress on the foot plantar tissue | Strong evidence for plantar pressure offloading efficacy and influence on comfort level | Common agreement on insole type and variations in practice according to pedorthists’ choices on casting methods where non-weight bearing and semin-weight bearing casting methods are most popular choices by the pedorthists. |
| Insole modification | Reduce peak plantar pressure  Reduce mechanical pressure and stress on the foot plantar tissue  Increase comfort level | Strong evidence for plantar pressure offloading efficacy and influence on comfort level | Common presenetation in practice according to pedorthists’ choices on removal of hard material and adding local cushioning, variations in choices of topcover replacements. |
