# Supplementary material for: Current practice in prescribing footwear and insoles to reduce the risk of neuropathic plantar forefoot ulceration and re-ulceration in people with diabetes
Source: PLoS One. 2026 Feb 10;21(2):e0341594. doi: 10.1371/journal.pone.0341594 (PMC12890116; doi:10.1371/journal.pone.0341594)
Supplement: S1 File — Survey questionnaire and the hypothetical cases. (DOCX) [file pone.0341594.s002.docx]

# Current practice in prescribing footwear and insoles to reduce the risk of neuropathic plantar forefoot ulceration and re-ulceration in people with diabetes

Appendix: Survey questionnaire and the hypothetical cases

The current practice of Australian pedorthists in prescribing footwear and insoles to prevent diabetic neuropathic ulceration and re-ulceration at the plantar forefoot

Q1 Thank you for your participation in this survey. Would you please fill out all questions as by cases that you would do in your everyday clinical practice mentioned below?

Q2 What is the post code of your primary pedorthic practice?

- ${loc://PostalCode (1) ________________________________________________

**Q3 Case-1:** Mary Smith is a 65 Years old female, Caucasian background, 86 kg weight, 170 cm height and very much full of life who lives in a privately owned home with her husband. She has a history of Type-2 Diabetes Mellitus (10 years+) and peripheral neuropathy (7 years+). Mary has recently healed a plantar ulcer under the hallux on the right foot. Bony prominence under 1st and 5th metatarsal heads (MTH's), bilaterally, Hammertoes and hallux abducto valgus (HAV), R>L, Hyperkeratosis on dorsal of 2 ^nd^ -3 ^rd^ interphalangeal joints (IPJ). Peripheral vascular disease and feet swell towards the end of the day. Initially, she was treated at a high-risk foot clinic and currently under community podiatry care. Mary does not qualify for state funding or NDIS and has private health insurance with top cover. She is also willing to pay the gap towards funding for her therapeutic footwear and insoles. What would you prescribe for her footwear and insole design and modifications? Please write your answer to this case in questions Q4-Q8.

**Q9 Case-2:** Reginald Bruce is 55 Years old male with, Australian Aboriginal background, 98 kg weight, 178 cm height, long term smoker and used to work as a social worker until recently. He is active and goes to the bush to collect his food. Reginald has a history of Type-2 Diabetes Mellitus (10 years+) and peripheral neuropathy (5 years+). He has recently healed plantar ulcer under the 3rd metatarsophalangeal joint (MPJ) on the left foot, osteomyelitis, tailor bunion and calluses on the lateral aspect of the 5th on the left.  Bilateral Hammertoes, R>L, Hallux amputation on Right foot (2 years ago). He also has rigid cavus feet. Initially, he was treated at a high-risk foot clinic and then discharged to community podiatric care; however, he has not attended for some time or no ongoing preventative care. His therapies are funded by the Closing the Gap program. What would you prescribe for his footwear and insole design and modifications? Please write your answer to this case in questions Q10-Q14.

**Q15 Case-3:** Suken Das is 70 Years old male with Fiji Indian background, currently on a disability pension and lives in community housing, currently not working. He gets carer support for three days/week. He is of 116 kg weight, 172 cm height, with a history of T2DM (18 years+) and peripheral neuropathy (12 years+). He has recently healed plantar ulcer at 1st metatarsophalangeal joint (MPJ) on the right foot, hallux limitus on the left.  He has significant oedema/ fatty tissue around his ankles, bilateral rigid flat foot,  trans-met amputation on left foot (3 years ago): nephropathy, hypertension. Initially was treated at a high-risk foot clinic and currently under community podiatry care. What would you prescribe for his footwear and insole design and modifications? Please write your answer to this case in questions Q16-Q20.

**Q21 Case-4:** Cathy Lee is 55 Years old female, and she is from an Asian background, currently on a disability pension. She is of 76 kg weight, 170 cm height, with a history of Type-1 Diabetes Mellitus (39 years+) and peripheral neuropathy (10 years+). She is a single mum and lives in her own home with her 30 Y/O daughter. Cathy is an artist and used to work as a volunteer at the local museum and local art gallery, but currently not very active. She has a recently healed plantar ulcer at 2 ^nd^ MPJ on Right foot. Over-riding digits 2^nd^ over 3 ^rd^ on Right, 2^nd^ and 3^rd^ toes amputated on the left (3 years ago), bony prominence and severe hyperkeratosis under 4^th^ and 5^th^ MTH on the left, Bilateral HAV. ATL (6 months ago), Rheumatoid Arthritis, retinopathy, at falls risk, Hyper-tension. Initially was treated at a high-risk foot clinic and currently under community podiatry care. What would you prescribe for her footwear and insole design and modifications?

Please write your answer to this case in questions Q22-Q26.

(Here is a sample of the questionnaire for each case which has been included in the Qualtrics survey for each case individually)

Q4 Footwear design parameters (please select as many parameters as applicable, describe them where appropriate and add any other recommended parameters in the additional boxes)

- Medical Grade Footwear without modification (Brand) (1) ________________________________________________
- Custom made footwear (2) ________________________________________________
- Medical Grade Footwear with modification (3) ________________________________________________
- Rocker sole design parameters (4) ________________________________________________
- Mid-sole materials (5) ________________________________________________
- Sole materials (6) ________________________________________________
- Upper materials (7) ________________________________________________
- Lining materials (8) ________________________________________________
- Re-lasting or widening (9) ________________________________________________
- Lace up (10) ________________________________________________
- Velcro fastening (11) ________________________________________________
- Additional parameters for shoe design 1 (12) ________________________________________________
- Additional parameters for shoe modifications 2 (13) ________________________________________________
- Additional parameters 3 (14) ________________________________________________
- Additional parameters 4 (15) ________________________________________________

Q5 Insole design parameters (please select as many parameters as applicable, describe them where appropriate and add any other recommended parameters in the additional boxes)

- Prefab insole (Brand) (1) ________________________________________________
- Custom made insole (2) ________________________________________________
- EVA Base (Hardness/Density) (3) ________________________________________________
- Tri-lam base (18)
- Poly Base (Thickness) (4) ________________________________________________
- Carbon Base (5) ________________________________________________
- Poron/PPT mid-layer (Thickness) (6) ________________________________________________
- EVA Mid-layer (Thickness) (7) ________________________________________________
- Plastazote topcover (Thickness) (8) ________________________________________________
- EVA Topcover (Hardness and Thickness) (9) ________________________________________________
- Leather topcover (10) ________________________________________________
- Adding additional arch support (11) ________________________________________________
- Metatarsal dome (Size and positioning) (12) ________________________________________________
- Metatarsal Bar (Size and positioning) (13) ________________________________________________
- Local deflection/removal of materials ( (14) ________________________________________________
- Local cushioning (15) ________________________________________________
- Additional parameters 1 for insole design (16) ________________________________________________
- Additional parameters 2 for insole design (17) ________________________________________________

Q6 What are the common challenges you may have with your recommendations and the patient's acceptance of them? How do you overcome those challenges?

- Possible Challenges (1) ________________________________________________
- Possible solutions to those challenges (4) ________________________________________________

Q7 How do you evaluate the offloading success?

- Clinical judgement based on experience (2)
- Ulcer recurrence (4)
- By In-shoe pressure measurements and analysis (5)

Q8 Any other observations or comments you may have about this patient's diagnosis and footwear, insoles prescriptions, please include below:

- Additional comments 1 (1) ________________________________________________
- Additional comments 2 (2) ________________________________________________
- Additional comments 3 (3) ________________________________________________
